# Food Insecurity among a Cohort of Division I Student-Athletes

**DOI:** 10.3390/nu14214703

**Published:** 2022-11-07

**Authors:** Jenifer Reader, Barbara Gordon, Natalie Christensen

**Affiliations:** 1 Department of Nutrition and Dietetics, Idaho State University, Pocatello, ID 83209-8117, USA; 2 Department of Nutrition and Dietetics, Idaho State University, Meridian, ID 83642, USA

**Keywords:** student-athletes, college athletes, Division I, food insecurity, food security

## Abstract

Background: Though the vulnerability of college students to food insecurity is well established, there is a paucity of studies focusing on the prevalence of food insecurity among student-athletes. Methods: A cross-sectional survey was conducted with collegiate athletes in the northwestern United States via an anonymous online survey. Food security status was assessed using the 10-item US Department of Agriculture Adult Food Security Survey. Results: Participating athletes (45/307, 14%) were primarily White, non-Hispanic (78%) females (73%) who lived and consumed meals off-campus (62% and 69%, respectively). Food insecurity was more prevalent among collegiate athletes than the general university population, 60% vs. 42%, respectively. Being a track or football athlete significantly predicted food security status (*p* = 0.002, *p* < 0.001, respectively). The risk for food insecurity was higher among collegiate football players (effect size, η^2^ = 0.86) compared with track athletes (effect size, η^2^ = 0.40). Conclusion: A statistically significant risk for food insecurity emerged among members of the football team. Factors contributing to disparate rates of food insecurity among college populations were explored and unique considerations for collegiate athletes discussed.

## 1. Introduction

College students are particularly vulnerable to food insecurity (FI), defined as insufficient access to enough nutritionally adequate food, acquired in socially acceptable ways, to support an active, healthy life [1,2,3,4,5,6]. In an analysis of 56 discrete college populations, prevalence rates ranged from 10% to 75%, with a weighted average of 41% [6].

There is a paucity of research, however, on the prevalence of FI among collegiate athletes. Six studies were found [7,8,9,10,11,12]. Three were published in the peer-reviewed literature [7,8,9], two in a database of dissertations [10,11], and the sixth in an online report from the Hope Center’s Longitudinal #RealCollege survey [12]. All examined FI through the lens of the school’s United States (U.S.) National Collegiate Athletic Association (NCAA) Division classification.

NCAA Division I is the highest level of collegiate competition; Division III is the lowest. Division I colleges are large schools and typically have more resources for student-athletes, e.g., fueling stations, also called re-fueling stations or training tables, which provide snacks and nutrition education to athletes before and after practice [11]. Division I and II schools are permitted to provide athletic scholarship funds; some scholarships include housing and meal plans, while others only pay for tuition. Division I and II schools also offer a “walk-on” recruitment option in which players are members of a university athletic team but do not receive scholarship funds [13]. Division III schools are not permitted to offer athletic scholarships [5] and do not offer walk-on recruitment [13].

Regardless of Division, collegiate athletes are under pressure to meet training and competition performance goals. This can be challenging for those consuming insufficient energy intake. Inadequate food intake can negatively impact muscle contraction, growth and repair, and cardiac function, leading to reduced levels of performance [14]. Furthermore, even when energy needs are met, inadequate intake of specific nutrients can lead to deficiencies and suboptimal performance or increased risk of injury and illness. Low iron intake, for example, can result in iron deficiency or iron deficiency anemia, and inadequate calcium and vitamin D can increase the risk of bone stress injury [14]. Additionally, student-athletes may experience Relative Energy Deficiency in Sport (RED-S), which refers to impaired physical and psychological functioning caused by inadequate dietary intake [15,16]. Multiple studies have also demonstrated that academic performance suffers among food insecure college students [3,7,17,18]. For NCAA Division I and II athletes, slipping academic performance can potentially put athletic scholarships at risk. FI can thus have a considerable impact on physical and mental health, as well as academic and athletic performance.

This study evaluated the prevalence of food insecurity among student-athletes at a 4-year Division I state university in the American northwest. The unique considerations for collegiate athletes are discussed. Factors contributing to disparate rates of FI among college populations are explored.

## 2. Materials and Methods

Employing a cross-sectional design and convenience sampling, data on demographics and food security status were collected. A 15-question online survey was constructed using Qualtrics software (Provo, UT, USA 2015). Five demographic questions queried respondents on gender, race/ethnicity, collegiate sport, living situation (on or off campus), and meal acquisition (university meal plan, self-purchased and prepared, or combination). The 10-item U.S. Department of Agriculture (USDA) Adult Food Security Survey (AFSS) was employed to determine food security status [19]. The AFSS asks about the adequacy of available food, access to healthy food items, meals skipped, and the impact of insufficient financial resources on food intake patterns [1]. The AFSS was not pilot tested for this study because the survey instrument has been previously utilized with college students [4,20]. The Institutional Review Board (IRB) declared the study protocol exempt.

Inclusion criteria were competitive athletes (≥18 years) who were active on a university sports team during the academic year 2021–2022. Athletes <18 years of age and/or without a current email on the active student-athlete university listserve were excluded. Table 1 details the complete list of inclusion and exclusion criteria.

To determine the sample size, a normal approximation to the hypergeometric distribution was calculated. The number of student-athletes meeting inclusion criteria (*n* = 307) was used as the population size. The population proportions (p and q) were set at 0.5, the confidence level at 95%, and the case z at 1.96. A sample size of 239 survey respondents was required to meet these parameters.

In April 2022, a one-time email was sent which included a link to the online consent form, providing details about the goal of the study, the type of information to be collected, how the data would be used, risks and benefits to participation, and other required information. Once participants confirmed their age (≥18 years) and agreed to the informed consent statement, students were directed to the anonymous, online survey. Survey directions requested participants respond based on their experience for the 2021–2022 academic year. Participants were offered the option to enter a drawing for a gift basket filled with non-perishable food items. To ensure anonymity, drawing data was stored separately from participant survey responses. The survey remained open for two weeks.

AFSS data were coded per instrument directions; specifically, “yes,” “often,” “sometimes,” “almost every month,” and “some months but not every month” were considered affirmative responses. Food security status was then stratified per USDA classifications—High, marginal, low, and very low food security [1]. High food security was defined as having no food access issues. In contrast, individuals who are marginally food secure experience one or two attributes of food insecurity, such as worrying about running out of food but with minimal impact on dietary intake. Low food security means an individual is struggling to consume a diet composed of a variety of nutrient-dense foods. Individuals with very low food security experience less than optimal intake of calories and periods of not eating for a day or more [1]. Given the small sample size, the four levels of classification were dichotomized into food secure (high/marginal food security) and food insecure (low/very low food security). In addition, a power analysis (one study group vs. population, dichotomous, alpha = 0.05) was conducted (ClinCal.com, Chicago, IL, USA).

Descriptive statistics were calculated for food security status and demographics (gender, race/ethnicity, language, housing situation, food sources [on-campus, off-campus, combination], collegiate sport). Three demographic variables (race, ethnicity, sport) and six categories of sports (basketball, golf, soccer, softball, tennis, volleyball) were condensed to provide meaningful interpretations. Frequencies for the combined variables were rerun.

Food security status was stratified by demographic variables. The Chi-Square Test was used to assess the bivariate associations between food security and demographic and sports variables. Linear regressions calculated the statistical significance of demographic variables on food security status. Statistical analysis employed SPSS version 27 (SPSS Inc., Chicago, IL, USA); significance was defined as *p* < 0.05, 95% Confidence Interval.

## 3. Results

Collegiate athletes responded to the survey over two weeks (15–27 April 2022). Ninety-four percent (45/48) of the respondents completed the survey. Three surveys were excluded from the analysis because of insufficient data; thus, 45 responses were analyzed. The findings reflect insights from 14% (45/307) of the total population of collegiate athletes at the university. A power analysis determined the sample size of 45 participants was powered at 70%.

About three-quarters (73%) of the participants were female; the majority were non-Hispanic/White (69%). Nearly two-thirds lived off campus (62%) and consumed meals off campus (69%). One respondent added the comment, “I used all the meals on my meal plan too early in the semester so now I eat off … stuff I make.” Most of the cohort were track athletes (44%), followed by 16% football players; 40% participated in other sports. Participant demographics are summarized in Table 2.

### 3.1. Prevalence of Food Insecurity

Among this cohort of athletes, nearly two-thirds (60%) reported being food insecure. Of note, 37% scored in the “very low” food security category. A smaller percentage of female athletes were food insecure compared with their male counterparts, 52% vs. 83%, respectively. All four of the respondents identifying as Hispanic were food insecure; in contrast, among the 33 athletes identifying as White/Non-Hispanic, about half (45%) were food insecure. The majority of food insecure athletes (55%) resided off campus. A significant bivariate association was found between collegiate sport and food security status (*p* = 0.048); however, Cramer’s V was 0.37, indicating a low effect size of the type of sport on food security status. See Table 3.

A linear regression evaluated the effect of participation in football and track on food security status; the sample sizes for the other sports were too small for analysis. Being a collegiate football player or track athlete significantly predicted food security status (*p* < 0.001, *p* = 0.002, respectively). The risk for food insecurity was higher for collegiate football players (effect size, η^2^ = 0.86) compared to track athletes (effect size, η^2^ = 0.40).

### 3.2. Food Security Challenges

Examples of food security challenges reported among this group of athletes included sometimes not getting enough food to consume a balanced diet, worrying about food running out, and food running out before acquiring the funds to replace it. More than half (51%) of the Division I student-athletes reported eating less often than they felt they should because there was not access to enough food. Furthermore, 73% reported that they did not eat for an entire day because of a lack of access to enough food for one or two months during the academic year.

## 4. Discussion

More than half (60%) of Division I student-athletes who participated in this study reported being food insecure. This percentage is higher than the general population; a cross-sectional study of the entire student body of the same university (*n* = 983) conducted in March 2020 (pre-coronavirus disease, COVID-19) found a 42% FI rate [4]. This finding is also higher than the results of other studies that evaluated FI among Division I athletes; rates ranged from 19% to 32% [7,8,12].

In this study, fewer student-athletes living on campus were food insecure, compared with those residing off campus (44.4% vs. 55.6%, respectively). This echoes the results of other studies, albeit the rates of FI were lower in the cohorts surveyed in those studies [7,8,12,21]. In one study, about 15% of participants identified living off campus with limited money as a major barrier to adequate food access [21]. A contributing factor to the higher FI rate among the student-athletes in this study may be considerable increases in regional off-campus housing costs. From 2019–2022, rent costs rose 28% [22]. To offset these increased housing costs in the setting of decreased scholarship funds received, some student-athletes at this university requested that a larger portion of their scholarship funds be allocated to housing and less toward meal plans [23].

A smaller percentage of student-athletes whose meals came fully from campus meal plans were food insecure (7.4% vs. 92.6%, respectively). Two other studies that evaluated FI among Division I athletes reported similar findings [7,12]. The disparities in the Brown et al. and Goldrick-Rab et al. studies, however, were less dramatic (11.5% vs. 29.9% and 21% vs. 37%, respectively) [9,12]. Brown et al. queried Division III collegiate athletes on the potential contributing factors to FI [9]. About one-fifth (18%) of athletes noted that their university meal plan funds were not sufficient to cover the quantity of food needed for a day or across a full semester. Nearly half (45%) stated that practice times conflicted with dining room hours; 22% with competition schedules. These barriers prevented them from fully utilizing their meal plans [9]. Of note, the latter reflects a trend reported by Van Woerden et al., who found that food insecure college students did not fully utilize their college meal plans [18].

Nearly three-quarters (73%) of the athletes in this study reported not eating for an entire day because of a lack of access to enough food for one or two months during the 2021–2022 academic year. This mirrors the findings of the Goldrick-Rab et al. report [12]. Half of the students in that study scored at the very low threshold for FI; thus, they were reducing their intake of food, skipping meals, and foregoing food for a day or more due to financial constraints [12]. Division I athletes must meet both academic and degree progression requirements while training and competing [12]. These academic mandates may result in busier schedules and interfere with mealtimes.

### 4.1. Unique Considerations for Food Insecurity Risk among Collegiate Athletes

In this study, the risk for FI was highest among football players. Nutritional requirements for football players are often higher than those for athletes in other sports, which may contribute to higher rates of FI among these student-athletes [24]. In addition, football is one of the largest teams on the campus in this survey. Thus, there is a greater number of football players with partial or no scholarship support. The lack of sufficient financial support may also be a contributing factor to FI. Race/ethnicity may also be a factor in the high rate of FI among football players. Nearly three-quarters (71%) of the football players who responded to the survey were Black/African American or Native Hawaiian/Pacific Islander. A large college health surveillance system (*n* = 13,720 students at 27 Minnesota state schools) reported a higher FI prevalence among both Black/African American and Native Hawaiian/Pacific Islander students; 43% and 36%, respectively [25]. Reeder et al. reported comparable results [25]. The Hope Center found that a higher percentage of Black/African American athletes are in NCAA football divisions [12].

Nearly half (44%) of respondents to this study were on the women’s track team. More females responded affirmatively to the FI questions compared with their male counterparts (17% vs. 10%, respectively). Like football, the large size of the track team translates to more track and field athletes receiving partial or no scholarship support. A survey of the general population of this university, however, did not find female students to be at significantly higher risk for FI compared to their male counterparts, as evidenced by an odds ratio of 0.81 (CI 0.60, 1.09, *p* = 0.17) [4]. Furthermore, a study conducted at a small Division III college in the northeastern U.S. at the start of the fall 2019 semester found that slightly more male than female athletes responded affirmatively to the FI questions, 32% vs. 27%, respectively [11].

At the time the survey was administered, as a COVID-19 safety precaution, the university fueling station was closed. The student-athletes surveyed, thus, had less access to free healthy snacks for refueling (e.g., peanut butter and milk). Furthermore, to accommodate athletes whose years of play were impacted by COVID-19, the NCAA allowed universities to permit athletes to play for additional seasons of competition and to carry additional members on scholarship on Division I sports teams [26]. Thus, schools allocated scholarship funds across a greater number of student-athletes, which likely decreased scholarship amounts received by each individual athlete.

### 4.2. Factors Contributing to Disparate Rates of Food Insecurity among College Populations

Several general factors may be contributing to the difference in the emerging FI prevalence rates among the studies on Division I student-athletes. Differences in FI rates appear to be influenced by the data collection timing (pre/post-COVID-19, semester), geographic area (rural/urban, region), and school classification (private/public).

This study surveyed students in April 2022, after the impacts of the COVID-19 pandemic. Davitt et al. found that college students experienced decreased levels of food security related to COVID-19, even if they were living off campus with parents [27]. Other studies which found lower FI prevalence rates among college athletes were conducted pre-COVID-19 (2016, 19% FI rate and 2018, 32% FI rate, respectively) [7,8]. The Goldrick-Rab et al. survey was administered before all of the financial consequences of COVID-19 escalated (2019, 26% FI rate) [12].

Data collection for this study occurred at a time indicative of the highest levels of college student FI; specifically, the end of the spring academic semester. An evaluation of the prevalence of FI among students at a northeastern U.S. college found lower levels of FI in the fall semester compared to the spring semester; 15% (*n* = 1123) and 20% (n = 1037), respectively [28]. Furthermore, surveying the same population of college students at the start and end of each semester, Bruening et al. found lower rates of FI at the beginning of each semester compared with the end; 35% vs. 28%, respectively [29]. Of note, Goldrick-Rab et al. and Poll et al. administered their surveys of college athletes at the start of the fall semester when rates of FI are at their lowest [8,12]. The Douglas et al. study spanned fall and spring semesters [7]. The inclusion of the spring semester data may explain why the Douglas et al. findings are higher than the Goldrick-Rab et al. and Poll et al. studies [8,12].

The athletes surveyed in this study attended a rural university in the northwestern U.S.—Both of these factors are associated with higher rates of FI among college students. Blagg et al. found higher rates of FI among students attending rural schools vs. those enrolled in universities in suburban and urban areas [30]. FI prevalence rates have also been found to be higher among college students in western and southern regions of the U.S., compared with northeast and midwest regions [30].

Of note, for students attending 4-year programs, FI rates have been found to be higher among those attending public colleges, compared with private schools [30,31]. The Douglas et al. and Poll et al. also surveyed Division I athletes attending public universities [7,8]. The Goldrick-Rab et al. study reported on the experiences of 3506 students at both private and public universities; the breakdown by classification was not provided [12]. The combination of both public and private universities in the study sample may explain the reason for the lower rate of FI discovered by Goldrick-Rab et al., compared with the Douglas et al. and Poll et al. studies and this study [7,8,12].

Table 4 highlights the factors which impact FI rates among both general college populations and student-athletes. The need for more rigorous research protocols is essential for determining an accurate assessment of prevalence among these populations.

### 4.3. Strengths and Limitations

A survey instrument (USDA AFSS) widely used with the college population was a strength of this study. Furthermore, survey directions instructed participants to respond based on their experience for the 2021–2022 academic year, eliminating the risk of data reflecting pre-college living scenarios and food security status. The incentive of a food basket, however, may have encouraged the participation of a greater percentage of FI athletes. The cross-sectional design and nonprobability sampling are also limitations. The small sample size and less than 80% power introduced the risk of a type II error. Though the homogeneity of the sample (44% Caucasian females) is a limitation of this study, 71% of female athletes in the NCAA are Caucasian [12]. Given the limited peer-reviewed, published data on FI among collegiate athletes, the findings of this research provide valuable insights. To better understand the dynamics underlying FI risk among student-athletes, future studies should also consider including questions about scholarships and meal stipends received by participants.

## 5. Conclusions

The prevalence of FI among Division I student-athletes was higher than in the general population of students at this public, rural, northwestern U.S. university. Balancing the competing demands of academic and athletic performance may put student-athletes at higher risk for FI. In this study, being a collegiate football player or track athlete significantly predicted food security status; FI risk was highest among collegiate football players. The timing of data collection (post-COVID-19, late spring semester) likely contributed to the higher rate of FI among participating athletes. The need for more rigorous research protocols is essential for determining an accurate assessment of the prevalence of FI among collegiate athletes.

## Figures and Tables

**Table 1:** Inclusion and Exclusion Criteria for Food Insecurity Survey Among Student-Athletes at Division I University in Northwestern U.S.

Inclusion Criteria	Exclusion Criteria
Active student-athletes on one of the university sports teams	Non-student-athletes
≥18 years of age	<18 years of age
Current email included on the active student-athlete university listserve	Current email not included on the active student-athlete university listserve
Active student-athletes enrolled during the 2021–2022 academic year	
All genders	

**Table 2:** Demographics of Participating Student-Athletes at Division I University in Northwestern U.S.

Variable		No. (%)
Gender	Female	33 (73.33)
	Male	12 (26.57)
Race/ethnicity	Hispanic	4 (8.89)
	White, non-Hispanic	31 (68.89)
	Other	10 (22.22)
Housing Situation	On-campus	17 (37.78)
	Off-campus	28 (62.22)
Food source	University meal plan	4 (8.89)
	Off-campus	31 (68.89)
	Off-campus/combination	10 (22.22)
Collegiate sport	Track	20 (44.4)
	Football	7 (15.6)
	Other sports	18 (40)

**Table 3:** Bivariate Comparison of Food Insecurity and Demographic Variables Among Student-Athletes at Division I University in Northwestern U.S.

	No. (%)			Odds Ratio	CI 95%	*p*-Value
	Total	Food Secure	Food Insecure			
All participants (*N* = 45)	45 (100.00)	18 (40.00)	27 (60.00)			
	Gender			4.706	0.891, 24.864	0.054
Female	33 (73.33)	16 (88.89)	17 (62.96)			
Male	12 (26.67)	2 (11.11)	10 (37.04)			
	Race/ethnicity			1.783	1.360, 2.337	0.213
Hispanic	4 (8.89)	0 (0)	4 (14.81)			
Non-Hispanic	41 (73.33)	18 (83.33)	23 (66.67)			
	Housing situation			0.481	0.134, 1.730	0.259
On-campus	17 (37.38)	5 (27.78)	12 (44.44)			
Off-campus	28 (62.22)	13 (72.22)	15 (55.56)			
	Food sources			6.382	0.200, 12.234	0.669
University meal plan	4 (8.89)	2 (11.11)	2 (7.40)			
Off-campus/combination ^a^	41 (91.11)	16 (88.89)	25 (92.60)			
	Collegiate sport			9.000	0.904, 86.612	0.048 ^b^
Track	20 (74.07)	12 (92.31)	8 (57.14)			
Football	7 (25.93)	1 (7.69)	6 (42.86)			

^a^ Combination = Consume meals via both meal plan and off campus ^b^ Cramer’s V = 0.37.

**Table 4:** Factors Impacting Food Insecurity Prevalence Rates Among College Populations in the U. S.

Year	FI Rate	Pre/Post COVID-19	Semester	Rural or Urban	Region of U.S.	Public or Private	1st Author
2016	19%	Pre	Fall, start	Urban	**SOUTHEAST**	**PUBLIC**	Poll [12]
2018	32%	Pre	Fall and spring	**RURAL**	**SOUTHWEST**	**PUBLIC**	Douglas [11]
2019	24%	Pre	Fall, start	**RURAL**	Northeast/West	**PUBLIC**	Goldrick-Rab [16]
2022	60%	**POST**	**SPRING, END**	**RURAL**	**NORTHWEST**	**PUBLIC**	Reader

Findings provided for research on FI among Division I athletes. Bolded capitalized font indicates a time when FI prevalence rates have been historically elevated.

## Data Availability

The data in this study are available on request from the corresponding author. The data are not publicly available due to the size of the database and privacy concerns.
